# Influence of BDNF polymorphisms on the neuronal‐enriched extracellular vesicle proBDNF/BDNF ratio and DMN activity in participants of the rrAD trial

**DOI:** 10.1002/alz.091552

**Published:** 2025-01-09

**Authors:** Pavel G. Yanev, Norman Scheel, Rong Zhang, David C Zhu, Ann M Stowe, Amanda Trout

**Affiliations:** ^1^ University of Kentucky, Lexington, KY USA; ^2^ Michigan State University, East Lansing, MI USA; ^3^ UTSW Medical Center/IEEM, Dallas, TX USA

## Abstract

**Background:**

Extracellular vesicles (EVs) are lipid bilayer nanoparticles (30‐10,000 nm) released from all cells that facilitate cell‐to‐cell communication. Cell type‐specific EVs can be enriched using cell‐specific surface markers. Neuronal‐enriched EVs (NEVs) contain measurable neurotrophins, pro‐ and mature brain‐derived neurotrophic factor (BDNF), that have opposing action in neuronal plasticity. Exercise shifts the proBDNF/BDNF ratio toward BDNF, potentially enhancing brain health. This study investigates the relationship between NEV proBDNF/BDNF, Default Mode Network (DMN), and a BDNF polymorphism (Val66Met) in the context of the NIH‐funded "Risk Reduction for Alzheimer’s Disease" (rrAD) trial (NCT02913664).

**Method:**

rrAD is a phase‐II randomized controlled trial with banked plasma and neuroimaging from 512 cognitively normal subjects (ages 60‐85, 34% aged 65‐85) with a family history of Alzheimer's Disease (AD) and/or memory complaints. The 372 subjects that provided DNA consent were genotyped for Val66Met (Rs6265). EVs were isolated using ExoQuick followed by anti‐NCAM‐L1 immunoprecipitation and analyzed for size/concentration with a Zetaview NTA. Levels of proBDNF/BDNF protein were quantified by ELISA in baseline, 12, and 24‐month plasma samples. MRI was performed on 3T scanners. Resting state functional (rs‐f) MRI data was pre‐processed using AFNI and ICA‐based strategy was used for noise reduction.

**Result:**

Of 372 consenting rrAD participants, 68% were Val/Val and 32% Met‐carriers (Val/Met and Met/Met). Of Met carriers, 56% were male (sex vs. Met‐carrier, P=0.33). A preliminary blinded cohort of EV isolation in n=21 subjects showed a substantial impact of BDNF Val66Met polymorphism on the proBDNF/BDNF ratio at baseline and 12 months. Higher BDNF in NEVs coincided with significantly stronger rs‐fMRI connectivity in the ventral and dorsal DMN (P =0.02). However, the precise nature of this relationship and how it relates to rrAD intervention responses remains to be fully understood.

**Conclusion:**

The study highlights the potential of BDNF polymorphisms in affecting the proBDNF/BDNF ratio and DMN activity, offering a valuable tool for disease prognosis and therapeutic effectiveness. It also suggests that AD trials need to account for BDNF polymorphisms to determine whether interventions aimed at slowing/preventing dementia are influenced by genetic factors and/or sex.

